# Time dependent dihedral angle oscillations of the spike protein of SARS-CoV-2 reveal favored frequencies of dihedral angle rotations

**DOI:** 10.1038/s41598-024-53954-9

**Published:** 2024-02-09

**Authors:** Oscar H. Bastidas, Zoran Sevarac

**Affiliations:** 1 Oscar Bastidas Research LLC, Minneapolis, MN USA; 2 Deep Netts Technologies LLC, RS, Belgrade, Serbia

**Keywords:** COVID-19, Spike protein, Dihedral angles, Therapeutics, Biophysics, Computational biophysics, Molecular biophysics, Diseases, Infectious diseases, Viral infection

## Abstract

The spike protein of SARS-CoV-2 is critical to viral infection of human host cells which ultimately results in COVID-19. In this study we analyzed the behavior of dihedral angles (phi and psi) of the wild-type spike protein over time from molecular dynamics and identified that their oscillations are dominated by a few discrete, relatively low frequencies in the 23–63 MHz range with 42.969 MHz being the most prevalent frequency sampled by the oscillations. We thus observed the spike protein to favor certain frequencies more than others. Gaps in the tally of all observed frequencies for low-abundance amino acids also suggests that the frequency components of dihedral angle oscillations may be a function of position in the primary structure since relatively more abundant amino acids lacked gaps. Lastly, certain residues identified in the literature as constituting the inside of a druggable pocket, as well as others identified as allosteric sites, are observed in our data to have distinctive time domain profiles. This motivated us to propose additional residues with similar time domain profiles, which may be of potential interest to the vaccine and drug design communities for further investigation. Thus these findings indicate that there is a particular frequency domain profile for the spike protein hidden within the time domain data and this information, perhaps with the suggested residues, might provide additional insight into therapeutic development strategies for COVID-19 and beyond.

## Introduction

As of March 2023, the World Health Organization reports that SARS-CoV-2, the pathogen responsible for COVID-19, has killed over 6.8 million people worldwide since the start of the pandemic^1^. This deadly virus infects the epithelial cells lining the respiratory tract by fusing its spike (S) protein with the Angiotensin Converting Enzyme 2 (ACE2) receptor on the host cell^2–4^. Although vaccines have been instrumental in curbing death rates, their efficacy has been observed to wane after 5–8 months and this, along with the rise of troubling new variants, creates a concern over the possibility of continuing infections of COVID-19^5^. Given the S protein’s critical role in the initial phases of infection, it is one of the most important drug targets and so understanding its architecture and mechanics remains a relevant research topic. The S protein is thus composed of three identical chains where each chain is made up of two subunits (S1 and S2)^2^. While the S2 subunit forms the fusion peptide, the S1 subunit is in turn composed of four domains: the N-terminal domain (NTD), C-terminal domain 1 (CTD1), C-terminal domain 2 (CTD2) and the receptor binding domain (RBD)^6,7^. Binding between the S protein and ACE2 thus begins with a conformational change of the RBD domain from the “down” position to the “up” position. Previous studies investigating various aspects of the S protein’s motions and mechanics include work that identified a latch region on the S protein that appears to regulate these “down” to “up” conformations^8^. Another study has identified centripetal motion of the RBD domain upon binding with ACE2 along with specific residues that played an important role in those motions^7^. Other work has taken the approach of considering the effects of dihedral angles associated with “down” to “up” conformational changes with another study obtaining insight into the entropy of residues most amenable to binding, by similarly looking at dihedral angles^9,10^. Although these approaches have yielded very important information in the fight against COVID-19, a dedicated characterization of the dihedral angles themselves is still lacking that specifically probes phenomena pertinent to time-informed dihedral angle fluctuations, such as, frequencies of oscillations and identifying potential dominant frequencies throughout the S protein. Consequently, the precedent currently remains modest for the questions posed by our work which motivates these present efforts.

For this work, the objective is to characterize the frequency domain of *steady-state* dihedral angle oscillations of the S protein. For this reason, time regimes such as the microsecond time-scale were avoided in order to preclude non-steady-state phenomena such as sudden domain motions and instead focus on dihedral angle dynamics at smaller time scales. The approach therefore consists of analyzing dihedral angle data (phi and psi), as a function of time, obtained from molecular dynamics simulations from our previous study of the trimeric S protein (PDB ID: 6VSB)^8^. The RBD domain of chain A for this structure is in the “up” position. For every residue, we analyzed frequency data obtained from the time domain to look for any frequencies that have a dominant/majority presence in individual chains, among amino acids (i.e. among all ARGs), as well as the whole protein. We also curated this data to see if there were any frequencies that uniquely persisted for any of the twenty amino acids throughout the simulation period. Lastly, we attempted to identify correlations between each residue’s motility in Euclidean space and its dihedral angle fluctuation variations. Phi and psi angle data are thus analyzed separately.

## Materials and methods

### Molecular dynamics

The trajectory of protein motions was obtained from previous work^8^ which used explicit solvent molecular dynamics simulations of the SARS-CoV-2 S protein using the NAMD2 program^11^ to carry out the simulation for a total of 200 nsec after equilibration with trajectories being written every nanosecond. Using CHARMM-Gui^12^, the protein was thus explicitly solvated with TIP3P water molecules and the CHARMM36m force field was selected. Missing residues in the experimental structure were added and disulfide bonds and glycosylated sites were also included. The simulation was carried out using the NPT ensemble which maintains the number of simulated particles, pressure and temperature constant. We used the Langevin piston method to maintain a constant pressure of 1 atm and we employed periodic boundary conditions for a water box simulation volume along with the particle mesh Ewald (PME) method with a 20 Å cutoff distance between the simulated protein and the water box edge. The integration time step was 2 femtoseconds and our simulation was conducted under physiological conditions (37 °C, pH of 7.4, physiological ionic strength with NaCl ions, LYS and ARG were protonated, HIS was not).

### Signal processing

Due to there being no sudden forces applied to the protein during the simulation that could result in steady state changes to the vibrational frequencies, for every residue, the Fourier transform was obtained from the phi vs. time and psi vs. time dihedral angle data (coming from the molecular dynamics simulations), using the fast Fourier transform algorithm from the JDSP library^13^ for the Java programming language (underlying calculation in Eq. 1). Positive absolute values of frequencies were thus calculated for the Fourier transform and the sampling frequency was once per nanosecond. This sampling frequency was deemed reasonable to avoid aliasing since information in the literature addressing protein dihedral angle timescales recognizes that, for example, chi dihedral angle changes associated with side chain rotations are on the order of nanoseconds^14,15^. Frequency domain data consequently spanned from 0 MHz to 496.094 MHz, just under the Nyquist/folding frequency of 500 MHz set by our fixed sampling rate (Nyquist frequency being half the sampling rate).

1 $$f^{o} \left( \omega \right) = \mathop \smallint \limits_{ - \infty }^{\infty } f\left( t \right)e^{ - i\omega t} dt$$

Equation 1: Fourier transform calculation to go from the time domain (*t*) to the frequency domain ($\omega$). *f(t)* represents the input time domain data (i.e. time domain data output from the simulation) to be transformed to the frequency domain. The exponential corresponds to the Fourier coefficients for representing the input function as a Fourier series. $f^{o} \left( \omega \right)$ represents the output function in the frequency domain.

### Data analysis

**Figure 1:**
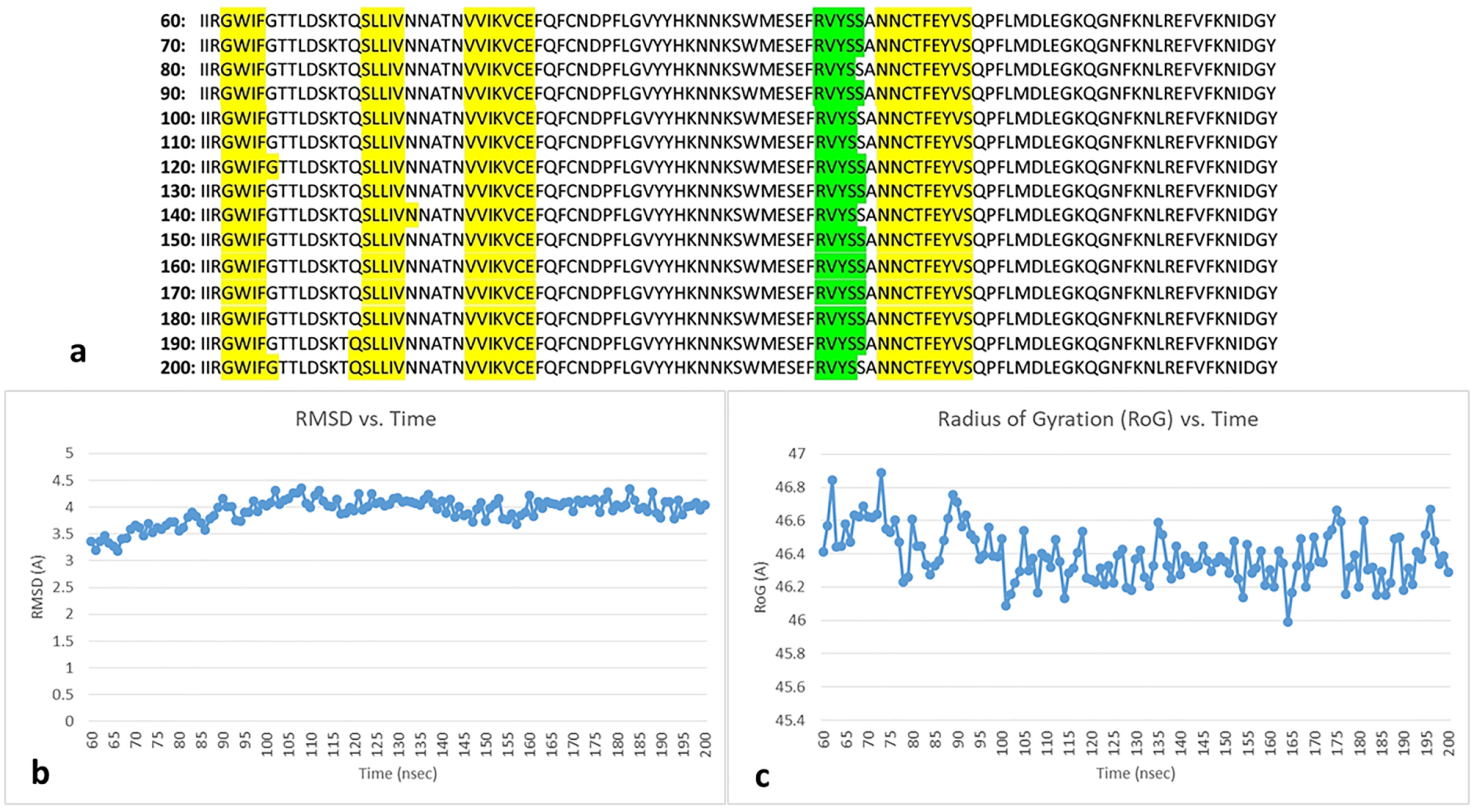
DSSP versus time, RMSD versus time and radius of gyration vs. time plots confirming steady-state regime of chain A. (**a**) DSSP versus time graphic of residues I100–Y200 for 60 nsec–200 nsec, for brevity, showing unchanging secondary structure over time (yellow highlights are beta strands and green highlight is alpha helix); data shown in increments of 10 nsecs, (**b**) RMSD versus time plot over the same 60 nsec–200 nsec displaying stable RMSD value over time, (**c**) radius of gyration vs. time for the same 60 nsec–200 nsec showing stabilized value over time (i.e. protein does not deform/change shape over time). Similar steady-state behavior was observed for chains B and C.

Steady-state regime of the protein’s fluctuations was confirmed by the Define Secondary Structure of Proteins (DSSP) algorithm, root mean square deviation (RMSD) and radius of gyration (RoG) metrics (Fig. 1)^16^. In order to identify frequencies that might be particularly abundant in the S protein, each time a peak appeared at a given frequency in the Fourier transform for an individual residue, it was tallied (the location of a peak at a given frequency in the individual residue’s spectrum being indicative that the dihedral angle possessed that frequency as a component of its oscillations). Peaks were identified as local maxima in the frequency domain data and so the DC component frequency at 0 MHz (typically a large value relative to the spectral peaks in our data) was disregarded. Such a tally was carried out as three independent analyses: 1) at the amino acid level, 2) at the individual chain level and 3) for the whole protein. Tally information was then plotted to visualize the abundance of the frequencies for each of the three cases. Next, root mean square fluctuations (RMSF) were calculated for every residue, and in order to quantify the tendency of dihedral angles to step outside of their respective mean (potentially a foreshadow to protein structural changes), population standard deviations were calculated for both phi and psi for each individual residue over the simulation period. These two metrics were then plotted (RMSF vs. phi/psi standard deviation) to see if there were any correlations observed between the two quantities. The standard deviations for phi and psi were then separately plotted as histograms to infer the spread of this variation. Lastly, in order to identify if any frequencies were unique to an amino acid (i.e. unique to, and persistent across all LYS in the protein), we carried out SQL inner table joins of the frequency data where peaks were specifically present, organized by amino acid (the table joins being responsible for identifying which frequency values were present across all the data for the amino acid being analyzed i.e. across all LYS). The complete data set is found in the electronic Supplemental Information material available at https://osf.io/n7tyg/.

## Results and discussion

**Figure 2:**
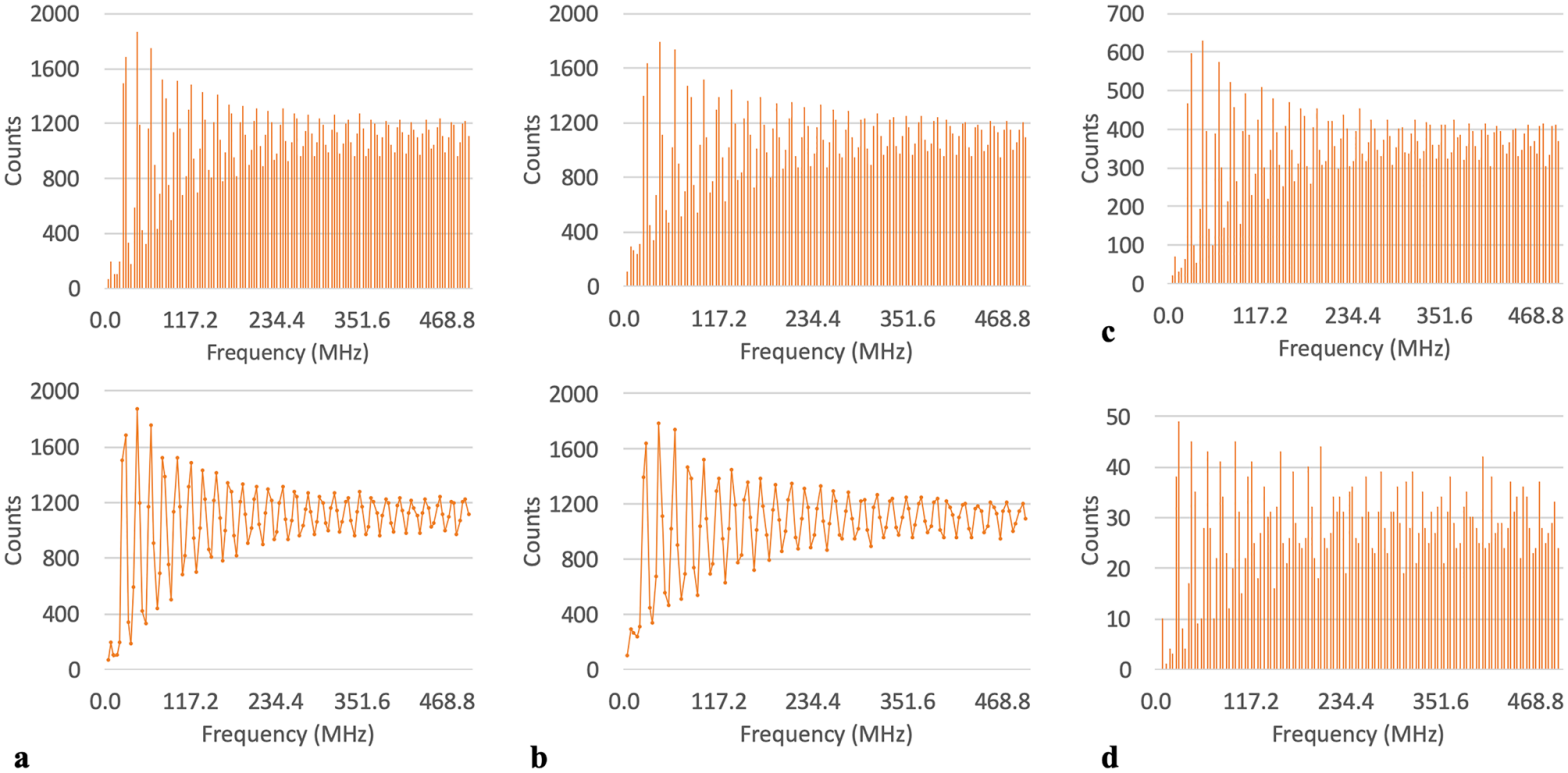
Tallied frequencies for all residues across all three chains for: (**a**) the phi dihedral angles showing bar chart in top panel and line plot in bottom panel (for ease in seeing profile), (**b**) the psi dihedral angle showing bar chart in top panel and line plot in bottom panel (for ease in seeing profile). (**c**) Tallied phi frequencies for all residues in the A chain and d) tallied phi frequencies for all threonines in the A chain (as an example of tally profile at the amino acid level).

**Figure 3:**
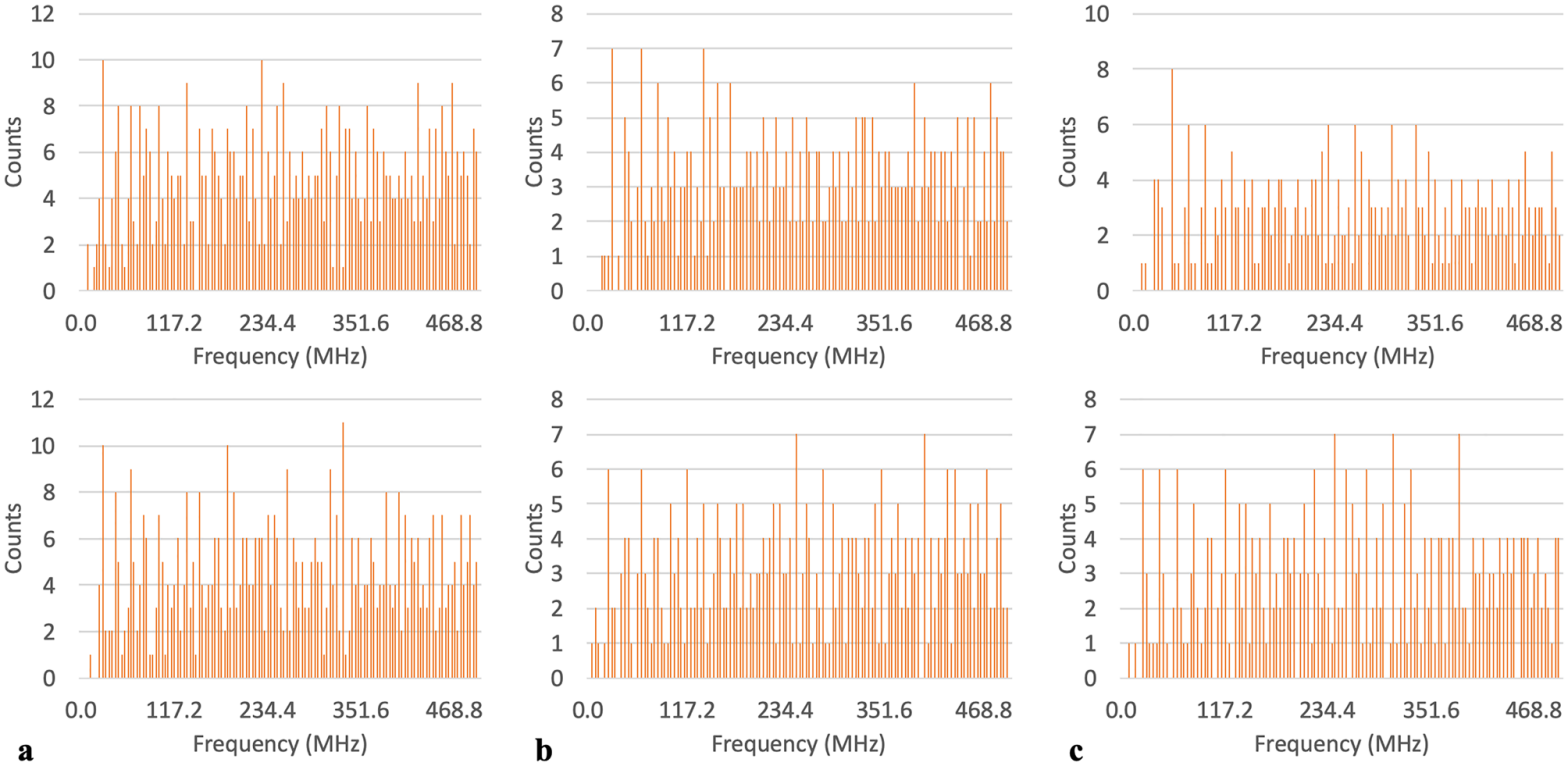
Frequency tallies showing un-accessed frequencies for: (**a**) all histidines in the A chain, phi and psi in the top and bottom panels respectively, (**b**) all methionines in the B chain, phi and psi in the top and bottom panels respectively, (**c**) all tryptophans in the C chain, phi and psi in the top and bottom panels respectively.

**Figure 4:**
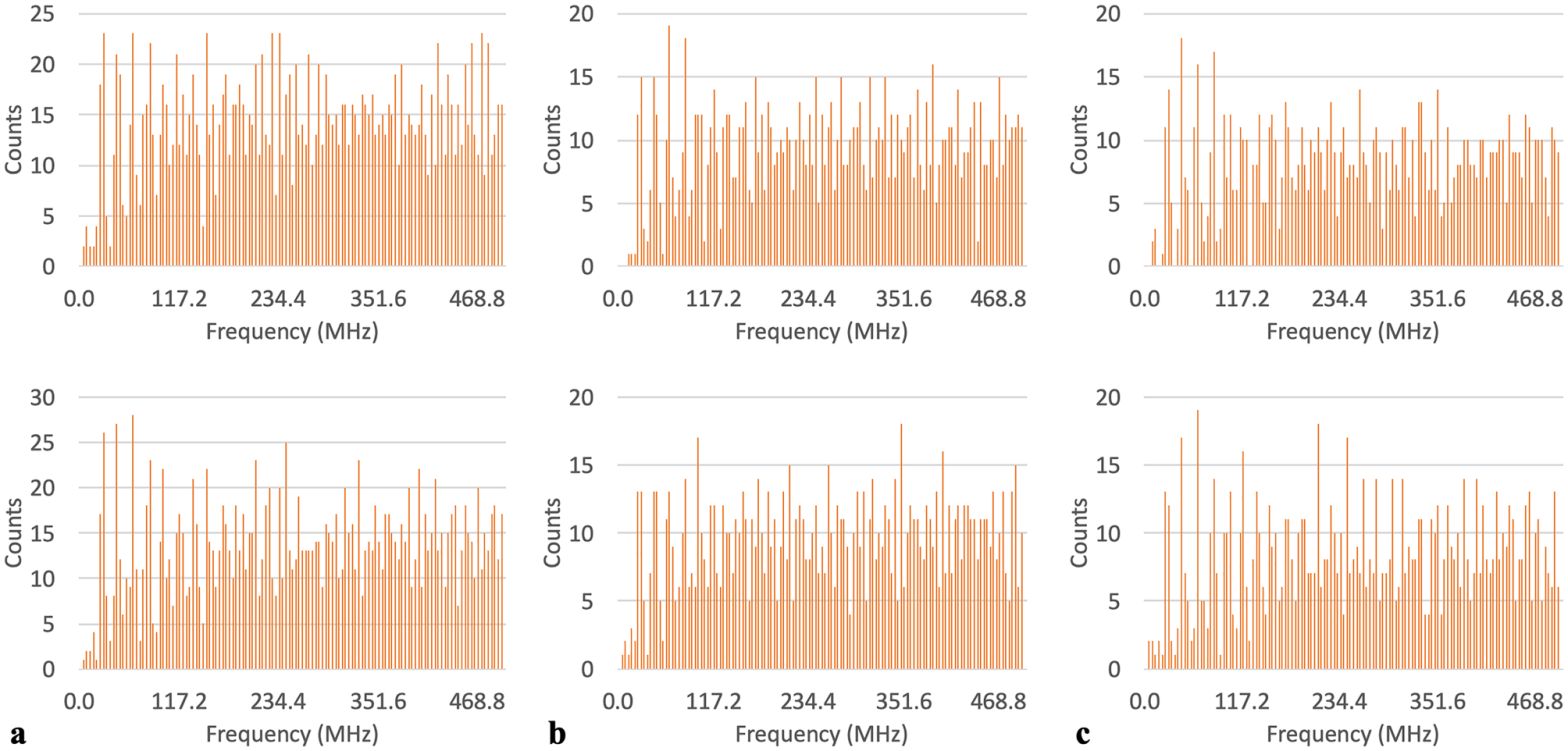
Tallied frequencies across all three chains for: (**a**) all histidines, phi and psi tallies in the top and bottom panels respectively, (**b**) all methionines, phi and psi tallies in the top and bottom panels respectively, (**c**) all tryptophans, phi and psi tallies in the top and bottom panels respectively.

Tallying all of the frequencies across the spectrum revealed that the most abundant frequency, throughout the entire protein, was 42.969 MHz, for both phi and psi dihedral angles, and the frequencies of 62.500 MHz and 27.344 MHz were observed as the second and third, respectively, most prevalent frequencies likewise for both phi and psi. Secondly, the profile of the plotted tally counts also showed that there was a regular alternation of high counts followed by low counts along the frequency spectrum and this difference in counts gradually became less pronounced as the frequency increased. This profile behavior was also observed for the plotted tally counts for just the individual chains, and to a lesser degree, for the amino acid level as well, within a given chain (see Fig. 2). The third observation was that for amino acids that had a relatively low abundance in the protein (HIS, MET and TRP), at the amino acid level within any given chain, it was observed that there were frequencies at which the dihedral angles (both phi and psi) never oscillated (see Fig. 3). There were between 9 and 15 of each of these three low abundance amino acids per chain, where each chain had a total of over 1000 residues thus establishing the low representation/abundance of HIS, MET and TRP in the protein. These un-accessed frequencies consequently appear as gaps in those plotted tallies. At the level of the whole protein (i.e. when tallies from all chains were combined), however, HIS in particular was observed to have populations of frequency counts that covered the whole spectrum for both phi and psi (Fig. 4). MET and TRP on the other hand still had un-accessed frequencies/gaps at the whole protein level for the phi dihedral angle, which were found at the lowest frequencies, but were able to cover the entire spectrum with psi (Fig. 4). Interestingly, despite the fact that the three constituent chains are identical in sequence, the un-accessed frequencies were not identical across the three chains.

**Figure 5:**
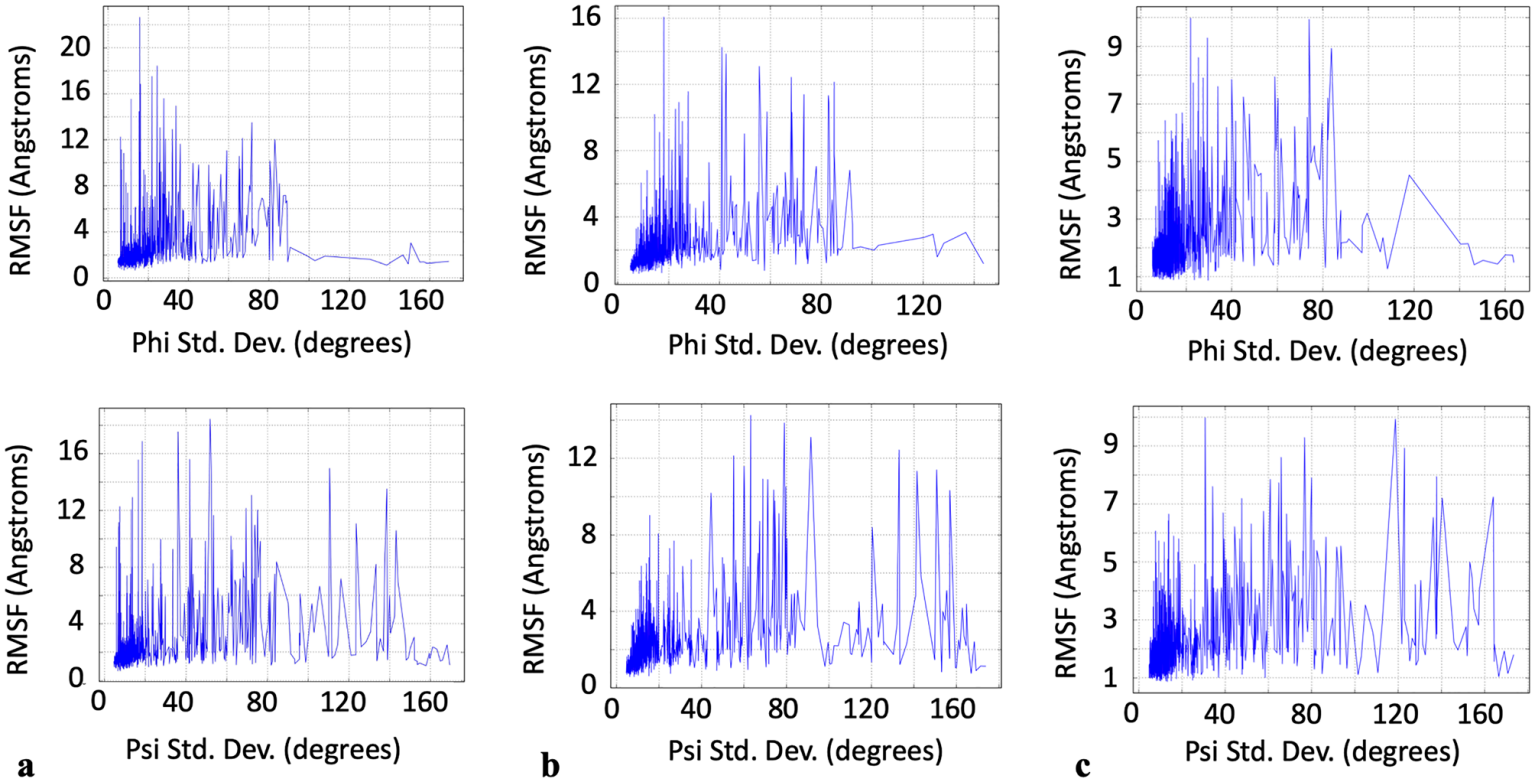
RMSF versus dihedral angle standard deviation for: (**a**) chain A, phi and psi in the top and bottom panel respectively, (**b**) chain B, phi and psi in the top and bottom panel respectively and (**c**) chain C, phi and psi in the top and bottom panel respectively.

**Figure 6:**
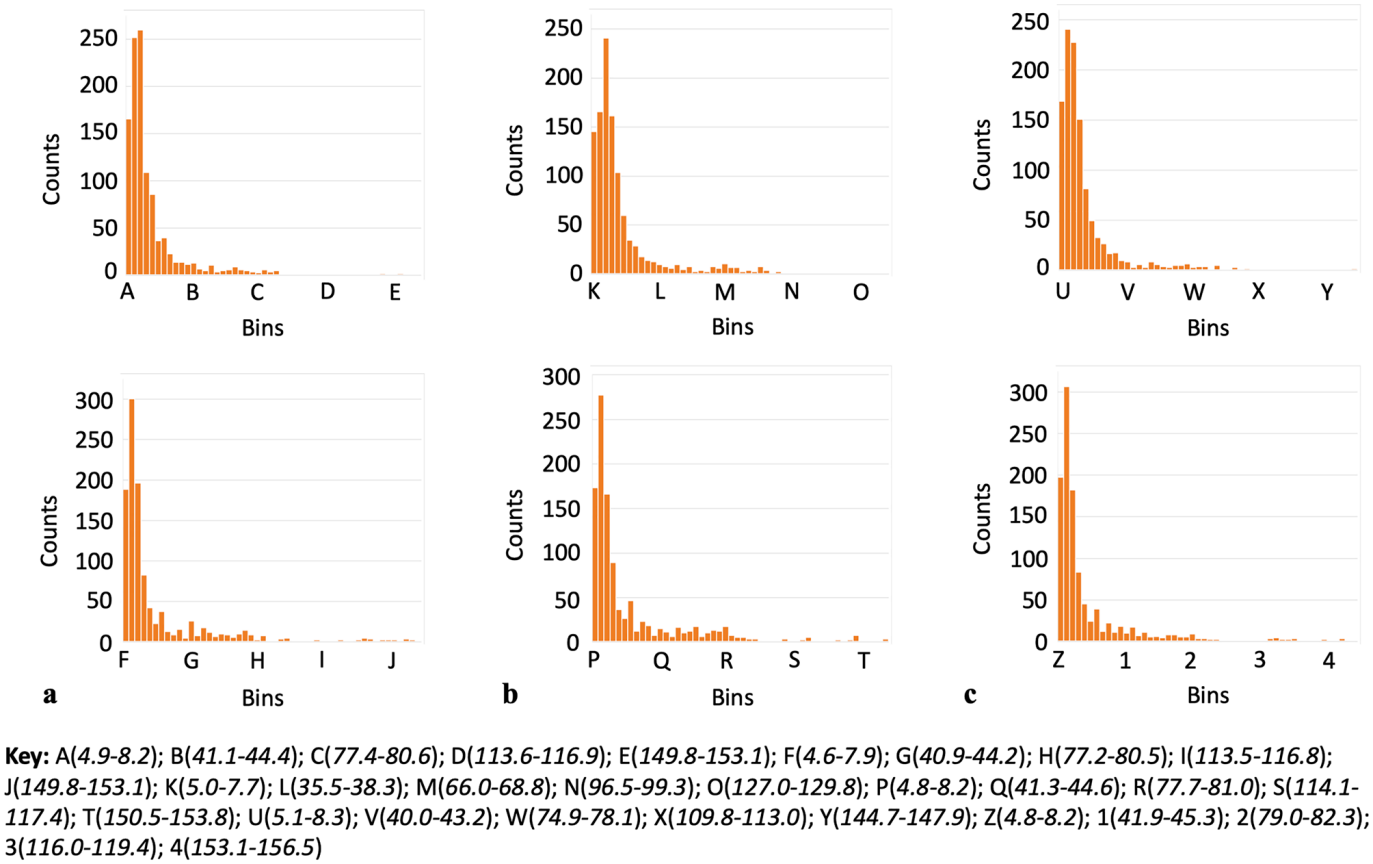
Dihedral angle standard deviation distributions showing positive skew profile for: (**a**) chain A, phi and psi in the top and bottom panel respectively, (**b**) chain B, phi and psi in the top and bottom panel respectively and (**c**) chain C, phi and psi in the top and bottom panel respectively. All histogram distributions done with 50 bins.

For each individual chain, plotting all of the residue’s RMSFs as a function of the standard deviations of their psi dihedral angles revealed that there was a larger quantity of residues with relatively large RMSF values corresponding to the larger values of the psi standard deviations (over 90°) compared to phi (Fig. 5). This was in contrast to the results observed for the phi standard deviations where there were fewer residues at these higher values of the phi standard deviations and what residues were present, they had relatively lower RMSF values (Fig. 5). The identity of the residues for both of these cases for standard deviations greater than 90° is thus shown in Table 1, specifically, for those residues with the top twenty RMSF values (phi, however, consistently did not have a full set of 20 amino acids at this upper range of standard deviations). Histogram inspection of the distribution of the values of the standard deviations, for both phi and psi, also revealed that both dihedral angles had a positive skew profile in their values (Fig. 6). Looking at each amino acid throughout each individual chain (i.e. all LYS in chain A, then in chain B, then chain C separately) revealed that there were no frequencies whatsoever that persisted across, or were unique to, any given amino acid throughout the simulation.

**Table 1:** Identity of residues with the top 20 RMSF values for dihedral angle standard deviations over 90° for: a) chain A phi, b) chain A psi, c) chain B phi, d) chain B psi, e) chain C phi and f) chain C psi.

a				b				c			
Amino Acid	Amino Acid #	RMSF (Å)	Φ Std. Dev	Amino Acid	Amino Acid #	RMSF (Å)	Ψ Std. Dev	Amino Acid	Amino Acid #	RMSF (Å)	Φ Std. Dev
GLY	891	3.073	150.726	SER	477	14.976	110.67	SER	256	6.825	91.248
THR	500	2.684	90.701	GLY	252	13.527	138.673	SER	98	3.537	92.306
GLY	89	2.01	146.756	GLY	482	11.073	123.635	GLY	550	3.076	136.751
PHE	1109	1.944	99.038	GLY	682	10.586	143.153	SER	591	2.972	123.962
SER	746	1.914	108.012	GLY	75	8.209	133.294	GLY	1099	2.765	120.257
GLY	593	1.627	130.43	GLY	485	8.066	137.636	GLY	548	2.422	128.144
VAL	227	1.505	103	GLY	257	7.493	138.83	GLY	107	2.295	102.508
GLY	550	1.457	169.528	LYS	150	7.194	116.148	SER	605	2.205	95.462
GLY	548	1.402	157.169	GLY	446	6.956	144.142	GLY	103	2.1	92.363
GLY	103	1.388	155.179	GLY	447	6.659	105.651	ASN	450	2.007	100.81
GLY	107	1.284	158.869	ALA	942	6.12	96.03	GLY	593	1.6	125.56
LEU	546	1.245	149.395	GLY	72	5.998	140.044	GLY	89	1.205	143.696
GLY	1059	1.141	138.717	ASP	442	5.474	90.189				
				THR	73	5.425	101.81				
				ASN	137	4.52	142.442				
				GLY	261	3.452	130.674				
				THR	333	3.406	102.933				
				PHE	1121	3.368	95.727				
				GLY	496	3.35	140.442				
				GLY	891	3.073	152.079				

d				e				f			
Amino Acid	Amino Acid #	RMSF (Å)	Ψ Std. Dev	Amino Acid	Amino Acid #	RMSF (Å)	Φ Std. Dev	Amino Acid	Amino Acid #	RMSF (Å)	Ψ Std. Dev
SER	683	13.111	91.494	GLY	1099	4.535	117.911	GLY	181	9.945	118.758
ASP	839	12.45	133.075	THR	941	3.208	99.492	LYS	150	8.934	122.996
GLY	842	11.405	150.773	GLY	381	2.786	97.382	SER	254	7.951	137.81
GLY	682	11.342	141.485	GLU	324	2.358	106.367	GLY	447	7.25	163.994
GLY	838	10.328	156.917	ASN	1125	2.313	91.4	GLY	446	7.211	140.348
GLY	257	8.405	120.45	GLN	836	2.206	90.607	GLY	75	6.543	136.219
ALA	27	5.835	143.333	GLY	103	2.147	143.795	GLY	184	6.339	141.429
GLY	181	5.09	155.467	ALA	520	2.138	140.49	HSD	625	5.555	93.595
GLY	184	4.842	140.915	GLY	891	1.863	105.18	ALA	626	5.522	91.693
GLY	72	4.507	114.191	THR	109	1.837	97.259	GLY	72	5.002	153.155
GLY	832	4.391	164.787	GLY	89	1.764	160.16	ALA	27	4.458	154.234
GLY	476	4.388	125.216	GLY	593	1.751	163.27	ALA	263	4.446	134.978
GLY	252	4.188	162.856	GLY	548	1.571	150.302	PHE	464	4.418	92.688
GLY	447	4.176	113.987	GLY	550	1.506	163.777	SER	256	4.372	130.514
GLY	446	3.987	153.751	GLY	107	1.445	156.637	GLY	838	4.224	127.916
GLY	75	3.539	154.669	GLY	1035	1.416	146.405	GLY	257	4.091	157.588
ASN	164	3.445	107.007	PHE	1109	1.28	108.362	GLY	842	3.931	134.412
GLY	1124	3.335	132.111					ALA	475	3.739	137.992
GLY	261	3.303	109.569					ALA	262	3.664	98.027
ARG	634	3.256	94.674					LYS	444	3.515	104.967

Phi angles did not have as many residues above a standard deviation of 90° compared to psi.

**Figure 7:**
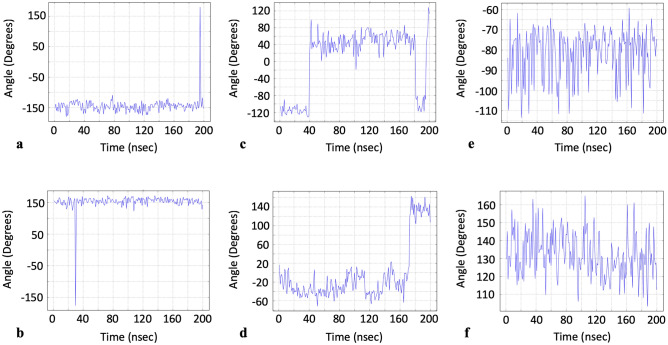
Dihedral angle values versus time for residues identified in literature as either important to allostery or the formation of a druggable pocket: (**a**) PHE 377^17^ chain C phi, (**b**) PHE 329^7^ chain B psi, (**c**) ASP 198^19^ chain C psi, (**d**) ASN 439^18^ chain A psi. Dihedral angle values versus time for most other residues: (**e**) PRO 39 chain A phi, (**f**) ILE 332 chain B psi.

**Figure 8:**
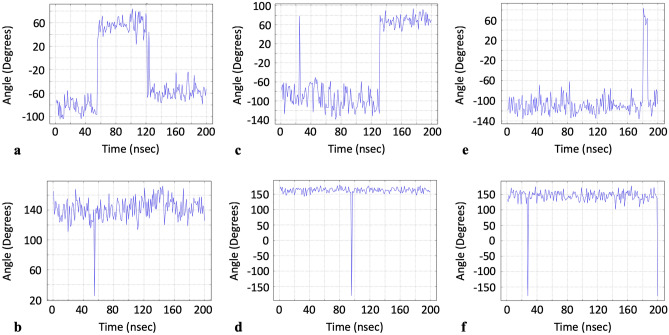
Dihedral angle versus time data for additional residues from our data set with time domain profiles similar to those residues previously identified as relevant to allostery and drug pocket formation: (**a**) ASN 211 chain A phi, (**b**) LEU 858 chain A psi, (**c**) THR 124 chain B phi, (**d**) THR 1077 chain B psi, (**e**) PHE 347 chain C phi, (**f**) PRO 82 chain C psi.

Several groups have previously identified residues important to either drug binding, allosteric effects, or both, for the S protein. According to work by Alvarado et al., LYS369 and PHE377 appeared to have important allosteric properties since binding of luteolin to these residues in the S protein induced an intense allosteric effect^17^. Verkhivker et al. reported that GLU406, ASN439, LYS417 and ASN501 were centers of allosteric interactions implicated in mediating long-range communications in the binding of the S protein with ACE2^18^. Of these residues, LYS417, ASN501 along with the additional residue of GLU484, were further identified by Verkhiker et al. to be important interacting centers that provide mutants at these positions with better binding affinity to ACE2. Xue et al. additionally identified PHE329 and PHE515 as residues critical to centripetal motions of the RBD domain upon binding with ACE2^7^. Drew and Janes also identified a druggable pocket and thus reported the amino acids lining this pocket^19^. Our own data showed that the dihedral angle vs. time profiles (phi, psi or sometimes both) for these very same residues, which were identified by the above previous groups, happened to have a distinctive profile that consisted of either a dihedral angle rapidly assuming a value far from the baseline, then immediately returning back to baseline, or the baseline would change very suddenly (Fig. 7). We note there were no distinctive frequency domain profiles for these residues, however. These unique time domain profiles were in contrast to those observed for most other residues whose dihedral angle values instead oscillated within a single fixed bandwidth of dihedral angle values (Fig. 7). Consequently, we present a list of some other residues which were identified in our dynamic data as having similar profiles, for further investigation as potential residues of interest to the drug design community (Fig. 8 and Table 2). Solvent accessible surface area (SASA) data for each residue is also provided to give preliminary insight into each residue’s potential accessibility to a drug-like compound (SASA data reported is for the conformation/trajectory-snapshot with the greatest SASA total for the whole protein). The complete list of suggested amino acids is in the electronic Supplemental Information. For completeness, we also present residues known to be important mutation sites which also happened to have distinctive time-domain profiles (Table 3)^18,20^. We also note that the above residues reported from the literature with distinctive time-domain profiles were not the same residues that had large dihedral angle standard deviations, suggesting that a high degree of angle rotatability does not necessarily foreshadow or correlate with allostery or pocket formation as originally speculated. Lastly, it is important to mention that although our workflow does not presume to replace existing methods to identify allosteric sites, binding pockets, etc.… our data might provide a motivation for possibly including dihedral angle time-domain data as a consideration in identifying allosteric sites, binding pockets, etc.…

**Table 2:** Additional residues with time domain profiles potentially indicative of some relevance to additional allostery or drug pocket formation.

Dihedral	Chain	Amino Acid	Amino Acid #	SASA (Å^2^)
Φ	A	ASN	211	106.838
	A	TYR	421	130.007
	A	SER	940	49.758
	B	THR	124	103.5
	B	PHE	400	4.699
	B	HSD	625	128.484
	C	SER	155	19.817
	C	PHE	347	31.224
	C	GLN	836	89.745
Ψ	A	THR	114	47.454
	A	LYS	444	187.497
	A	LEU	858	24.479
	B	ALA	93	10.51
	B	GLN	493	70.155
	B	THR	1077	7.32
	C	PRO	82	47.145
	C	MET	177	122.114
	C	GLY	757	4.043

SASA data reported in Angstroms squared.

**Table 3:** Residues important to SARS-CoV-2 mutations with distinct time-domain profiles.

Dihedral	Chain	Amino Acid	Amino Acid #	Mutation
Φ	B	ALA	570	ASP
	C	ASN	501	TYR
Ψ	A	GLU	484	LYS
	B	LYS	417	ASN
	C	PRO	681	HIS

## Conclusions

Given the importance of the S protein as a drug target for COVID-19, in this study, we characterized the oscillatory behavior of the protein’s phi and psi dihedral angles in order to identify the dominant frequency components of oscillations. The findings suggest that, for the SARS-CoV-2 spike protein: 1) both phi and psi dihedral angles are dominated by a few discrete relatively low frequencies in the 23–63 MHz range with 42.969 MHz being the most prevalent frequency sampled by the oscillations, 2) the protein appears to possess some sort of “harmonic series” type property with the frequencies sampled, as observed by the alternating high-low–high profile of the tallied frequencies suggesting the protein is more “in tune” with some frequencies compared to others (we refer here to the harmonic series of acoustics), 3) frequency components sampled by dihedral angles may be a function of position in the primary structure since amino acids in relatively low abundance in the protein never oscillate at certain frequencies in contrast to relatively abundant amino acids (i.e. the more positions an amino acid is found in, the more frequencies it can sample), 4) sampled frequencies also appear to be impacted by the micro-environment from non-bonded atoms since even though the three protein chains are identical in sequence, un-accessed frequencies for low-abundance amino acids were not identical across the three chains, possibly due to the chains’ spatial orientation differences given one chain is in the “up” position, 5) highly motile residues in Euclidean space also appear to have a wider degree of psi angle rotatability as observed by the positive correlation between high RMSF values and large standard deviation values for psi compared to phi and 6) specific residues identified in the literature as constituting the inside of a druggable pocket as well as other residues identified as allosteric sites, or important mutation sites are observed in our data to have distinctive time domain profiles. This last observation motivated us to propose other residues from our dynamic data with similar time domain profiles, which could possibly be associated with additional pocket formation or allostery phenomena since it has been observed that druggable pockets, for example, are dynamic and transient in nature^21^. Naturally, future dedicated work would be needed to confirm the significance of our proposed residues as well as whether distinct time-domain profiles are correlated with allostery, mutation sites, etc.… for generalized cases. In all, our present work therefore suggests that there is structured behavior hidden in the seemingly random time domain dihedral angle fluctuation data of the S protein. We postulate, however, that many of these observations were likely made possible due to the large size of the protein which permitted a wide sampling of frequency components in the dihedral oscillations for both phi and psi. Nevertheless, further studies are needed to confirm if this behavior is universal to all proteins, regardless of size, or if it is unique to the SARS-CoV-2 S protein. Additional studies involving a plethora of different proteins with a wide size distribution would also be needed to determine the physical significance of the most prevalent frequency components (high or low) as they relate to protein size and composition, for instance. We also note that additional studies would be needed to identify if the frequency domain (such as the DC component in our data) is impacted by actual physics in the system, such as the effects of the quantity of ions and water and temperature, or if phenomena, such as the DC component, are purely artifactual (we call to mind how vertical measurements due to gravity in accelerometers such as those found in smartphones can cause large DC components in those systems as well). Our present work would thus serve as a reference for such a future study.

## Data Availability

The datasets generated during the current study are available in the Open Science Framework repository, https://osf.io/n7tyg/.
